# Predators of monarch butterfly eggs and neonate larvae are more diverse than previously recognised

**DOI:** 10.1038/s41598-019-50737-5

**Published:** 2019-10-04

**Authors:** Sara L. Hermann, Carissa Blackledge, Nathan L. Haan, Andrew T. Myers, Douglas A. Landis

**Affiliations:** 1 Department of Entomology, The Pennsylvania State University, University Park, USA; 2 Department of Entomology, Michigan State University, East Lansing, USA; 3 Program in Ecology, Evolutionary Biology and Behavior, Michigan State University, East Lansing, USA

**Keywords:** Ecology, Behavioural ecology, Conservation biology

## Abstract

Conserving threatened organisms requires knowledge of the factors impacting their populations. The Eastern monarch butterfly (*Danaus plexippus* L.) has declined by as much as 80% in the past two decades and conservation biologists are actively seeking to understand and reverse this decline. While it is well known that most monarchs die as eggs and young larvae, few studies have focused on identifying what arthropod taxa contribute to these losses. The aim of our study was to identify previously undocumented predators of immature monarchs in their summer breeding range in the United States. Using no-choice feeding assays augmented with field observations, we evaluated 75 arthropod taxa commonly found on the primary host plant for their propensity to consume immature monarchs. Here we report 36 previously unreported monarch predators, including representatives from 4 new orders (Orthoptera, Dermaptera, Lepidoptera and Opiliones) and 11 taxa (Acrididae, Gryllidae, Tettigoniidae, Forficulidae, Anthocoridae, Geocoridae, Lygaeidae, Miridae, Nabidae, Erebidae and Opilliones). Surprisingly, several putative herbivores were found to readily consume immature monarchs, both in a targeted fashion or incidentally as a result of herbivory. This work expands our understanding of the monarch predator community and highlights the importance of unrecognized predation on insects of conservation concern.

## Introduction

Reports of declining insect populations globally have spurred widespread concern and prompted dramatic headlines^1–3^. Declines are likely to be linked to multiple causes, including habitat loss and fragmentation, pesticides and other forms of pollution, invasive species, climate change, and others^4–6^. Declines in Lepidoptera are particularly well documented^6–8^, in part due to their charismatic appearance and relative ease of identification. Within this group, immature life-stages—especially eggs and neonates—are particularly vulnerable and frequently experience high mortality rates that average around 40% but can exceed 95%^9^. Several abiotic and biotic factors contribute to these early losses, but predators can account for a large proportion of this mortality^10,11^. Therefore, understanding interactions between Lepidoptera and their predators can be an important step toward managing species of conservation concern.

The monarch butterfly (*Danaus plexippus* L.) is among the most widely recognised insects worldwide, notable for its abundance, interactions with milkweed host plants (Gentianales: Apocynaceae) and its migratory behavior^12^. The Eastern migratory monarch population undertakes an annual long-distance migration from breeding grounds in the eastern United States (US) and Canada to overwintering grounds in central Mexico. However, this population has declined at an alarming rate since the mid-1990s^13^, suggesting the migratory phenomenon is in peril^14^. Multiple hypotheses have been advanced to explain this decline, including loss of overwintering habitat^13,15^, threats along the migration routes^16^, and loss of milkweed host plants from the summer breeding range^17,18^. However, causes of the decline are likely multifactorial, and significant effort is being put into identifying other factors that might contribute to monarch declines including climate change^19^, altered disturbance regimes and differential predation among host habitats^20,21^.

One hypothesis suggests the loss of common milkweed (*Asclepias syriaca* L.) from the summer breeding range could be responsible for much of the decline^17,18,22–25^. For example, common milkweed (hereafter milkweed) prevalence in parts of the Midwestern US has declined by as much as 90% from 1999–2009 following the widespread adoption of herbicide resistant corn and soybean and subsequent use of broad-spectrum herbicides^26^. Accompanying the loss of milkweed in the summer breeding range is a shift in the habitat where remaining milkweed stems occur. With the near elimination of milkweed from annual crop fields, most remaining monarch breeding habitat in the Midwestern US occurs in perennial grasslands^27,28^. Previous studies show that grasslands also support large and diverse populations of arthropod predators^29^ and predation of Lepidoptera eggs in these habitats frequently exceeds those in annual croplands^21,30^. Therefore, the effect of milkweed loss from croplands in the Midwestern US on monarch declines may be exacerbated by the potential for increased risk of predation in remaining grassland habitat^20^.

Like many Lepidoptera, survival of monarch eggs and early-instars is quite low^31^. Some mortality is due to interactions with milkweed defenses^32^ or extreme weather events^33^, but predation is also a key factor in monarch mortality^31^. One study reported 78% mortality of monarch eggs and 59% mortality of first instars, and noted that on occasion ants removed 100% of eggs and larvae from individual plants^34^. Another study reported approximately 98% mortality of sentinel monarch eggs after 7 d and high rates of loss on plants with ants and aphids present in a Wisconsin, US old-field^35^. A study conducted in Minnesota, US considered cumulative proportion survival of monarchs in a restored prairie and found only 20% of eggs survived to hatching, with <10% survival to 2nd instar, and <2% to 3^rd^ instar^31^. However, despite this evidence, predation was seldom directly observed and most eggs and young larvae were reported to simply disappear.

To more fully understand the impact of predation on monarchs, we need information on which arthropods contribute to the loss of eggs and young larvae. In 2015, a literature review identified 12 arthropod taxa as predators of monarch eggs and/or larvae^36^; these included members of the following taxa: Chrysopidae^36^, Formicidae^31,35,37–40^, Coccinellidae^34,41,42^, Araneae^31,37,43^, Vespidae^43,44^, Pentatomidae^31,43^ and Mantidae^45^. However, many other arthropods are commonly found on milkweeds, causing us to ask if other taxa also contribute to predation losses. Finally, while many of the milkweed-specialist herbivores have been studied in detail^46–49^, the wider community of generalist herbivores and omnivores that frequent milkweed stems has received less attention. To our knowledge, no one has directly examined the potential for these arthropods to consume monarchs; they could do so intentionally, or incidentally while eating leaf material where monarch eggs or larvae are present.

Given the likely importance of predation in limiting monarch population growth, the aim of this study was to identify which of the many arthropods that visit common milkweed have the potential to prey on monarch eggs and young larvae. We used field observations to determine which arthropods visit milkweed, then tested their propensity to consume monarch eggs and neonate larvae in laboratory no-choice trials. Finally, we used additional observations to confirm predation under field conditions. Based on previous observations, we predicted that most predatory and omnivorous taxa would consume eggs and larvae, and that at least some of the putatively herbivorous taxa would do so as well.

## Results

We collected a total of 779 individual arthropods from 75 taxa across 11 orders and 33 families and tested their predation potential under no-choice laboratory conditions. From these we found 34 unique taxa representing 8 orders which consumed monarch eggs and 30 taxa across 8 orders that consumed neonate monarchs (Table 1). These include 4 orders of arthropods not previously reported to consume immature monarchs (Orthoptera, Dermaptera, Lepidoptera, and Opiliones), including 11 new families and 25 species (excluding at least 11 other distinct taxa not identified to the species level). Monarch eggs were consistently consumed (defined here as predation in >50% of trials with n ≥ 3) by 16 taxa including: *Melanoplus differentialis* (Thomas) and other Acridide spp., several *Oecanthus and Allonemobius* species, *Nabis americoferus* (Caryaon), *Podisus maculiventris* (Say)*, Neoconocepjalus spp*. and other katydids in the family Tettigoiidae, Chysopidae, *Forficula auricularia* (Linnaeus), *Plagiognathus spp*., *Coleomegilla maculata* (DeGeer), *Crematogaster cerasi* (Fitch) and *Tapinoma sessile* (Say). Monarch neonates were consistently consumed by 11 taxa including: several *Oecanthus and Allonemobius* species, *N. americoferus*, adult *P. maculiventris*, Chysopidae larvae, *F. auricularia*, various Coccinellidae and Formicidae (Table 1). We also found 22 taxa that consumed eggs or neonates occasionally (at least once but in less than 50% of trials, or with n < 3), and 9 taxa that consumed monarchs in every trial but which were observed with very limited replication (n < 3).

**Table 1:** Milkweed visiting arthropods which consumed monarch butterfly eggs and/or neonate larvae in 48 h laboratory no-choice tests.

Order	Family	*Genus species*	Common names^a^	Eggs^b^	Larva^b^
**Orthoptera**	**Acrididae**	***Melanoplus femurrubrum***	**redlegged grasshopper**	2/6	0/7
		***Melanoplus differentialis***	**differential grasshopper**	6/11*	3/16
		***Melanoplinae***	**spur-throated grasshopper (I)**	1/5	1/3
		**Various spp**.	**grasshopper adults**	4/6*	—
	**Gryllidae**	***Oecanthus nigricornis spp. group***	**blackhorned tree cricket group**	1/1	1/1
		***Oecanthus nigricornis spp. group***	**blackhorned tree cricket group (I)**	6/11*	3/4*
		***Oecanthus niveus***	**narrow winged tree cricket**	1/1	1/1
		***Oecanthus fultoni***	+**snowy tree cricket**	1/1	—
		***Allonemobius allardi***	**Allard’s ground cricket**	3/3*	1/1
		***Allonemobius fasciatus***	**striped ground cricket**	3/3*	3/3*
		***Allonemobius*** **spp**.	**cricket**	—	1/1
	**Tettigoniidae**	***Neoconocephalus*** **spp**.	**common conehead (I)**	1/1	—
		**Various spp**	+**katydid (I)**	2/3*	—
Neuroptera	Chrysopidae	**Various spp**.	**green lacewing**	0/2	2/5
		Various spp.	+green lacewing (I)^25^	11/11*	4/4*
**Dermaptera**	**Forficulidae**	***Forficula auricularia***	+**European earwig**	8/10*	13/14*
Hemiptera	**Anthocoridae**	***Orius insidiosus***	**insidious flower bug**	2/7	2/7
	**Geocoridae**	***Geocoris*** **spp**.	**big-eyed bug**	0/1	1/1
	**Lygaeidae**	***Lygaeus kalmii***	+**small milkweed bug**	1/4	0/1
	**Miridae**	***Plagiognathus spp*** *.*	+**black mirid bug**	15/19*	0/2
		***Lygus spp*** *.*	**Lygus bug**	2/11	0/8
		***Adelphocoris lineolatus***	**alfalfa plant bug**	—	1/3
	**Nabidae**	***Nabis americoferus***	**common damsel bug**	6/9*	6/6*
		***Nabis subcoleoptratus***	**black damsel bug**	—	1/1
		***Nabis spp*** *.*	+**damsel bug**	1/1	—
		***Nabis spp*** *.*	**damsel bug (I)**	—	1/1
	Pentatomidae^20,26,32^	*Podisus maculiventris* ^45^	+spined soilder bug	0/4	5/5*
		*Podisus maculiventris*	+spined soilder bug (I)	7/10*	8/10*
Coleoptera	Coccinellidae^23,30^	***Coccinella septempunctata***	**sevenspotted lady beetle**	1/12	2/15
		***Coleomegilla maculata***	**pink spotted lady beetle**	6/9*	6/9*
		***Cycloneda munda***	**polished lady beetle**	3/8	3/3*
		*Harmonia axyridis* ^31, 42^	+multicolored Asian lady beetle	8/10*	10/10*
		*Harmonia axyridis*	multicolored Asian lady beetle (I)	8/8*	11/11*
		***Hippodamia convergens***	**convergent lady beetle**	1/5	2/5
		***Hippodamia parenthesis***	**parenthesis lady beetle**	1/4	0/1
		***Hippodamia variegata***	**variegated lady beetle**	0/10	1/9
		***Propylea quatuordecimpunctata***	**14 spotted lady beetle**	5/12	3/4*
Hymenoptera	Formicidae^20,24,26–29^	***Crematogaster cerasi***	**acrobat ant (colony)**	4/4*	2/5
		***Formica subsericea***	**+** ***Formica subsericea*** **(colony)**	1/4	0/1
		***Formica vinculans***	***Formica vinculans*** **(colony)**	1/2	5/7*
		***Tapinoma sessile***	**odorous house ant (colony)**	4/8*	0/6
		***Tetramorium caespitum***	**pavement ant (colony)**	3/20	3/11
**Lepidoptera**	**Erebidae**	***Euchaetes egle***	**milkweed tussock moth (I)**	2/4*	0/3
Araneae^20,26^	Aranidae	Various spp.	orb-web spiders	0/28	5/22
	Salticidae	Various spp.	+jumping spiders	0/23	7/19
	Thomosidae	Various spp.	+crab spiders	1/34	7/26
**Opiliones**			**+harvestmen**	0/7	3/7

Taxa reported as predatory on monarchs for the first time are in bold, those that consumed monarchs in 50% or more of the trials are followed by*. Common names preceded by + indicate taxa confirmed to consume monarch eggs or larvae in the field. Fractions indicate number of individuals consumed/number of replicates performed. ^a^Adults were tested unless indicated by (I) = immature stage tested. Ants were tested as colonies. ^b^Number of individuals that consumed eggs or larvae/total number tested. Since field-observed predators were not always identified to the same taxonomic resolution as those in the lab, some observations are noted at higher taxonomic levels (e.g., Chrysopidae, Opiliones). Superscript bracketed numbers represent previously published studies in which taxa were listed as predatory on immature monarchs.

Independent field observations confirmed that many of the potential predators identified by our lab trials also prey on monarch eggs and/or neonates under natural field conditions^50^. These include: *Oecanthus fultoni* (Walker), species in the family Tettigoniidae, and Chrysopidae, *F. auricularia*, *Lygaeus kalmii* (Stål), *Plagiognathus spp.*, *P. maculiventris*, a Nabidae, *Harmonia axyridis* (Pallas), *Formica subsericea* (Say), and two families of arachnid, Salticidae and Opilliones. In addition, individuals in the following families that we did not test in lab trials were observed to consume monarch eggs or larvae in the field: Carabidae, Cantharidae, and Trombididae.

The number of milkweed-visiting taxa that did not eat monarch eggs and or larvae in our trials was approximately equal to the number that consumed them (Table 2). These include several taxa that have previously been reported as monarch predators (e.g., *Tenodera aridifolia sinensis*, *Polistes dominulus*), or are part of known predaceous groups (e.g., Pentatomidae, Coccinellidae and Formicidae).

**Table 2:** Milkweed visiting arthropods which did not consume monarch butterfly eggs or neonate larvae in 48 h laboratory no-choice tests.

Order	Family	*Genus species*	Common names^a^	Eggs^b^	Larva
Orthoptera	Acrididae	*Melanoplinae*	spur-throated grasshopper	0/2	0/1
	Tettigoniidae	Various spp.	katydid	—	0/2
Mantodea	Mantidae	*Mantis religiosa*	European mantid	0/2	0/3
		*Mantis religiosa*	European mantid (I)	0/2	0/1
		*Tenodera aridifolia sinensis* ^34^	Chinese mantid	—	0/1
Hemiptera	Anthocoridae	*Orius insidiosus*	insidious flower bug (I)	—	0/3
	Lygaeidae	*Oncopeltus fasciatus*	large milkweed bug	0/3	0/3
	Pentatomidae	*Cosmopepla lintneriana*	twice-stabbed stink bug	0/1	0/6
		*Euschistus variolarius*	onespotted stink bug	—	0/1
		*Thyanta spp*.	stink bug (I)	—	0/1
		*Trichopepla spp*.	stink bug (I)	—	0/1
	Alydidae	*Alydus eurinus*	broad-headed bug	0/1	0/6
	Reduviidae	*Sinea diadema*	spined assassin bug	0/2	0/2
		*Sinea diadema*	spined assassin bug (I)	0/1	—
		*Phymata pennsylvanica*	Pennsylvania ambush bug	—	0/1
	Rhopalidae	*Harmostes reflexulus*	scentless plant bug	0/1	0/1
	Rhyparochromidae	*Ligyrocoris diffusus*	dirt-colored seed bugs	0/2	—
		Various spp.	seed bug	0/1	—
Coleoptera	Cantharidae	*Chauliognathus pensylvanicus*	goldenrod soldier beetle	0/8	—
		*Chauliognathus marginatus*	margined leatherwing	0/1	—
		*Polemius canadensis*	soldier beetle	—	0/3
	Carabidae	*Calleida punctata*	carabid beetle	0/1	—
	Cerambicidae	*Tetraopes tetrophthalmus*	red milkweed beetle	0/9	0/20
	Chrysomelidae	*Labidomera clivicollis*	swamp milkweed leaf beetle	0/4	—
		*Paria thoracica*	leaf beetle	0/3	—
		*Criocerinae*	leaf beetle	—	0/1
	Coccinellidae	*Brachiacantha ursina*	ursine spurleg lady beetle	0/4	0/1
		*Coccinella septempunctata*	sevenspotted lady beetle (I)	0/1	—
		*Coleomegilla maculata*	pink spotted lady beetle (I)	0/2	—
		*Propylea quatuordecimpunctata*	14 spotted lady beetle (I)	0/2	—
	Lampyridae	*Photinus indictus*	no lantern photinus	0/1	—
		*Photinus pyralis*	big dipper firefly	0/2	—
		*Photinus spp*.	various photinus	0/2	0/7
	Lycidae	*Calopteron reticulatum*	banded net-wing beetle	0/1	—
	Scarabaeae	*Popillia japonica*	Japanese beetle	0/9	0/1
Diptera	Dolichopodidae	Various spp.	long-legged fly	0/2	—
	Stratiomyidae	*Nemotelus kansensis*	soldier fly	0/1	—
Hymenoptera	Formicidae	*Lasius neoniger*	turfgrass ant (colony)	0/7	0/2
	Vespidae^23,33^	*Polistes spp*.	vespid wasp	0/1	—

^a^Adults were tested unless indicated by (I) = immature stage tested. Ants were tested as colonies. ^b^Number individuals that consumed eggs or larvae/total number tested. Superscripts represent previously published studies in which these taxa were listed as predatory on immature monarchs.

## Discussion

The decline of the Eastern migratory monarch overwintering population has sparked concern from citizens and scientists alike. While many factors likely contribute to the decline, predation is one of the most significant sources of mortality for eggs and neonates^31^, and may be exacerbated by the monarch’s increased reliance on perennial grasslands where predator populations are diverse and abundant^20,29,30^. A recent modeling study predicts that as little as a 4% increase in survival of breeding monarchs in the North Central US could potentially lead to recovery of the overwintering population^24^; therefore, understanding which arthropods prey on monarchs is an important step toward designing and managing monarch-friendly habitats.

There are several reports of monarch predation in the literature; however, most of these are anecdotal or based on stochastic events which do not capture the breadth of potential predators in milkweed habitats. In addition, all predation events to-date have been observed in daylight hours yet a recent study suggests that a significant portion of predation occurs at night^50^, further suggesting current observations are lacking. In the most complete list of monarch predators prior to our work, the majority of observations focused on predators of adults and only 12 predators of monarch eggs and neonates were described^36^. Our results more than double the number of predators of immature monarchs and show they are far more diverse than previously reported. We found 30 new egg predators and 25 new larval predators, including representatives from 11 families and 4 orders that were not previously reported to prey on monarchs. Despite the monarch being a classic example of defense sequestration leading to protection from higher trophic levels we still see a considerable amount of predation^51,52^. Such findings highlight the importance of evaluating the breadth of predation for this insect and the many other specialists thought to have escaped such top-down interactions by commandeering host plant defenses.

Ants have long been implicated as important egg and larval predators of monarchs, and are common on milkweed plants^34^. For example, in one recent study they comprised 69% of all predatory arthropod individuals found on milkweeds in grasslands^20^. In another, *Formica montana* (Wheeler) removed all sentinel monarch eggs in ca. 90 min in a grassland setting^35^. Fire ants (*Solenopsis invicta* Buren) have also been implicated as a driver of immature monarch mortality in Texas^38,39^, and in other studies ant abundance was negatively related to immature monarch survival^40^. Many of the ant species we tested also fed on monarchs, although they differed in their behaviours and preferences. For example, *Tetramorium caespitum* occasionally removed both eggs and larvae, while *Tapinoma sessile* removed eggs but not larvae, and *Formica vinculans* (Wheeler) removed one of two eggs but attacked larvae aggressively and consistently. To allow ants to forage naturally in our trials, we gave entire colonies access to arenas with monarch eggs or neonates. However, we observed that some colonies foraged much more actively than others and removal rates are in part a function of colony activity levels. We also noted interesting differences in foraging behaviour. For example, *T. sessile* only foraged nocturnally, removing eggs in 4 out of 8 trials. In contrast, in the acrobat ant *C. cerasi* foraged diurnally in large groups and removed eggs in all trials, sometimes within a just a few minutes of gaining access to the container. However, *C. cerasi* removed only 2 of 5 larvae despite frequently encountering and antennating them. Finally, *Lasius neoniger* (Linnaeus) which has been reported as a voracious predator of other Lepidopteran eggs in turf grass^53^ did not attack eggs or larvae in our trials.

The presence of spiders on milkweed has also been associated with increased monarch mortality^31^. In our experiments, we tested three spider families: Aranidae (orb-web spiders), Salticidae (jumping spiders) and Thomosidae (crab spiders). All three families were found to consume monarch neonates, but only spiders in the family Thomosidae consumed monarch eggs (and did so in only 1 of 34 trials). Spiders may have favoured neonates over eggs because they often rely on prey movement in their foraging behaviour^54^. Since spiders are abundant and diverse in grassland habitats, a larger survey of spiders including ground-dwelling taxa which were not tested here could provide additional clarity on the role of these predators in monarch mortality.

Individuals from both Forficulidae and Coccinellidae are commonly observed on milkweed plants and may be important predators of monarch eggs and neonates^21^. In our study, *F. auricularia* were consistently predaceous, consuming eggs in 8 out of 10 trials and neonates in 13 out of 14 trials. *F. auricularia* are predominantly nocturnal and in the field we often observed them resting during the day hidden in the newly forming leaves at the apex of milkweed plants, a position also favored by 1^st^ and 2^nd^ instar monarch larvae^31^. In addition, we tested 9 species of Coccinellidae; of which, the adult forms of all species except *Brachiacantha ursina* (Fabricus) consumed monarch eggs or larvae. In contrast, immature Coccinellidae were generally less likely to consume monarchs, although replication for this life stage was low. We note that while we tested taxa from 8 Coleopteran families, Coccinellidae were the only family to consume monarchs. In particular, *H. axyridis* was the most consistent predator from the family Coccinellidae in our trials. In the larval form they consumed eggs and neonates in all trials (n = 8 and 11, respectively); adults consumed eggs in 8 of 10 trials and neonates in 10 of 10 trials. It has been previously demonstrated that *H. axyridis* is capable of imposing strong predation pressure on immature monarchs in controlled laboratory and field trials^42,55^. However, when *H. axyridis* were presented with alternative aphid prey (*Aphis nerii* Boyer de Fonscolombe), monarch consumption declined with increasing aphid populations^55^. Additional studies in open-field settings will help to elucidate the role of lady beetles on immature monarch survival.

In addition to known predatory or omnivorous taxa, we also examined the consumption potential of several common, but putatively herbivorous, arthropods. While not previously reported as monarch predators, all 12 of the Orthopteran taxa we tested consumed monarch eggs or neonates, and some did so quite consistently (although for Melanoplinae and individuals from the family Tettigoniidae, results differed between immature and adult stages). Interestingly, most of the incidences of predation by these herbivores occurred without herbivory, i.e. they selectively removed eggs directly off the foliage without consuming the foliage itself. In other instances, Acrididae individuals and *Euchaetes egle* (Drury) ate eggs along with the foliage they consumed. At least one previous study has documented monarch eggs removed from plants as a result of plant defoliation by insect and non-insect herbivores^43^. While the frequency of herbivores encountering monarch eggs and neonates in the field is unknown, some milkweed specialists (especially, *E. egle* and *L. kalmii*) can have large populations and may frequently encounter monarch eggs in the field. Finally, some species we tested remained strict herbivores. For example, *Tetraopes tetrophthalmus* (Forster) and *Popillia japonica* (Newman) failed to consume eggs or larvae in all trials.

In our assays, we used a no-choice laboratory arena to test for predation potential and consequently the behaviors we documented may not be representative of a field setting. Prey acceptance may increase under no-choice conditions due to starvation and increased encounter rates^56^. For example, in the field a given predator may forage on a different part of the milkweed plant than where monarch eggs or neonates are typically found, or might prefer alternative prey. In a prior study *Polistes* dominulua (Christ) readily consumed monarch larvae in no-choice assays, yet when provided with a choice between the toxic late-instar monarch larvae and a less toxic *Pieris rapae* (Linnaeus) or *Trichoplusia ni* (Hübner) they preferentially consumed the alternate prey^44^. Likewise, some arthropods that are important predators in a field setting might not eat monarchs in the artificial environment of a lab-based no-choice trial, and would need to be discovered using other methods. Two of the predators we tested (*Tenodera aridifolia sinensis* Saussure, *Polistes spp*.) which did not consume monarchs in our assay were previously reported as predators in the literature^36^. It is possible that the no-choice arena used in our assays simply did not facilitate normal foraging for these species. For these reasons it is best to confirm lab results against field observations. In this regard, representative species from eight of the nine orders we found to consume monarchs in lab no-choice tests were independently observed to prey on those stages under field conditions (Table 1). Additional field observations are needed to determine if the other taxa we observed to consume monarchs in the lab also do so in the field.

Evidence suggests most monarch eggs and neonates in summer breeding habitats succumb to predation^31,35^, and predation may be more prominent as monarchs now use milkweed in grassland habitats where predator abundance and diversity is high^29^. Therefore, in addition to filling gaps in the natural history of a well-studied organism, identifying monarch predators could provide knowledge that proves useful to conservation efforts. Reducing predator prevalence in important monarch habitats, or prioritizing habitats where predation pressure is lower, could allow more monarch eggs and neonates to reach adulthood and help to stabilize the overwintering population.

## Methods

We tested a wide range of arthropod taxa found on milkweeds to determine whether they would consume monarch eggs and/or larvae. For each arthropod we began with the null hypothesis that it would not consume monarchs of either life stage. Any observation of predation during the feeding trials caused us to consider that taxon a potential predator and to seek confirmation that they attack monarchs under field conditions.

### Collection and identification of potential predators

We limited the range of arthropods we tested to those observed or collected on common milkweed, since they are most likely to encounter immature monarchs in a field setting. We excluded potential aerial predators not found foraging on milkweed stems, as well as parasitoids^36,57^. Arthropods used in the experiment were field collected from *A. syriaca* patches using sweep nets, aspirators, or hand collection in Ingham County, Michigan, USA and State College, Pennsylvania, USA during the summers of 2017 and 2018 and used in trials within 24 h of field collection. Once trials were completed, arthropods were frozen, placed in 70% ethanol, and identified to the lowest possible taxonomic level. In a few cases arthropods were only identified to coarse taxonomic groups (e.g., family or genus); this was particularly true for spiders and immature stages of some orders. Therefore, some test groups could potentially contain multiple species, and we refer to the group as “various spp.” To be conservative, we count each of these groups as a single predatory taxon even though it is possible it contains more than one species.

### Egg predation trials

**Figure 1:**
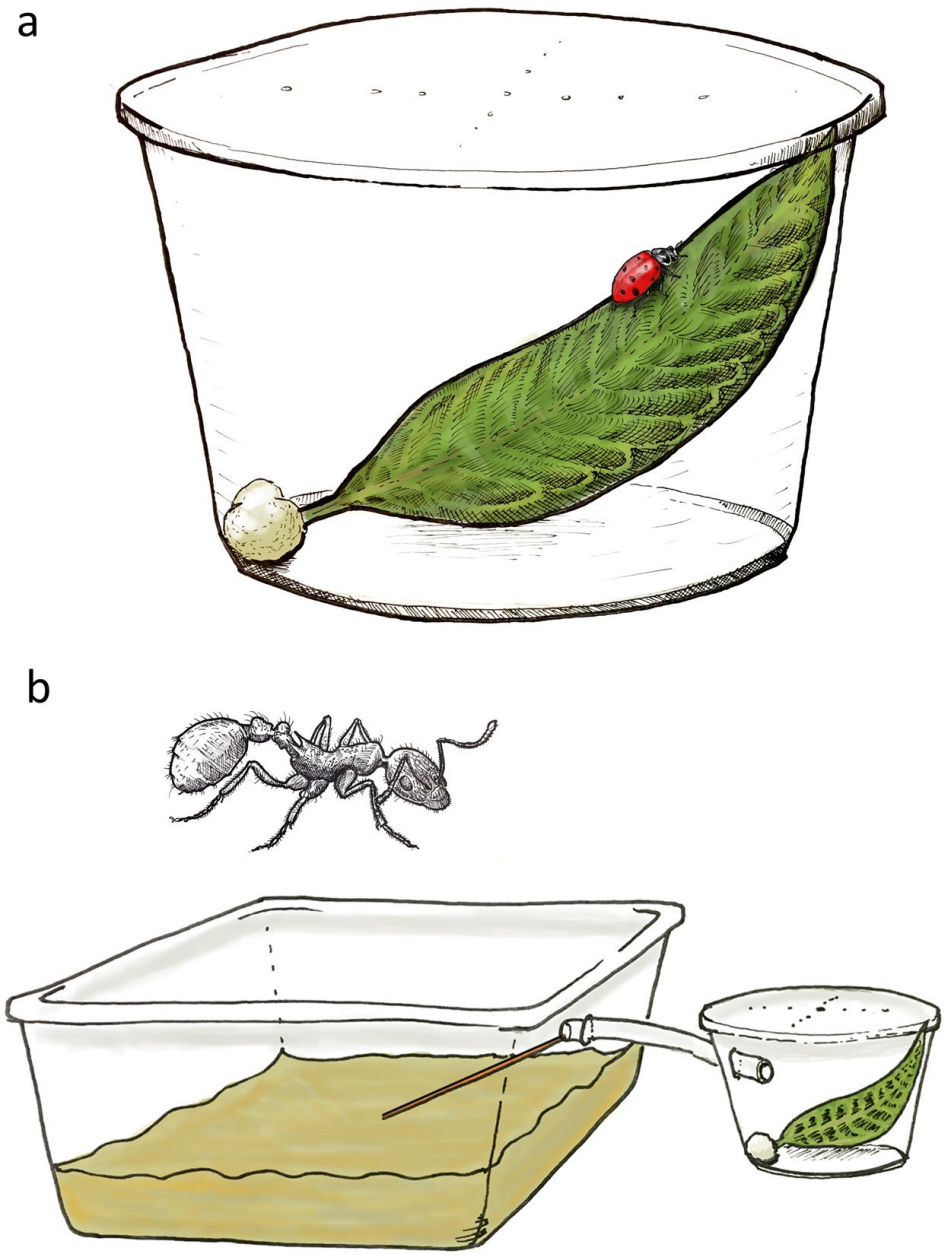
No-choice arenas used to test whether arthropods would consume monarch eggs or neonates. (**a**) Setup for most trials, in which the arthropod was placed on a milkweed leaf in a deli cup arena, with either a monarch egg or neonate for 48 h. (**b**) Setup for testing the predation potential of ants. Ant colonies housed in plastic containers were connected to arenas using clear tubing; ants accessed the tubing via a removable bridge. Top edges of colony containers and arenas were coated with fluon to prevent ants from escaping.

To determine which milkweed-visiting arthropods can consume monarch eggs, we performed no-choice assays in 473 mL (16 oz.) deli-cup arenas (Solo Bare DM16R-0090). Larger predators (e.g., Mantodea and Opiliones) were placed in 946 mL (32 oz.) deli-cup containers (Solo Bare DM32R-0090) to allow for more natural movement. Field collected leaves of common milkweed were rinsed in tap water to remove any naturally occurring arthropods or debris. Late in the season when common milkweed was senescing, swamp milkweed (*Asclepias incarnata* L.) leaves of the same general shape and size were field collected and used in trials. Leaves of approximately 10 cm in length were placed diagonally against the side of the deli-cup arena to allow potential predators full access to forage on the top and bottom of leaf surfaces (Fig. 1). The petiole of each leaf was placed in a damp cotton ball to avoid desiccation during the experiment. A single monarch egg, obtained from a colony of wild monarchs, was lightly glued (Elmers^®^ Glue-All) to the bottom side of each leaf, with a fine tipped paintbrush, to mimic the natural placement of eggs in nature. Field trials confirmed that naturally foraging predators readily consumed eggs attached in this fashion^21^. A single predator was placed in each deli-cup; then the cup was sealed with a perforated lid. Cups were placed in a climate-controlled growth chamber at 27 °C and 16:8 light cycle. Egg presence/absence was recorded at 48 h and each egg was also examined under a dissecting microscope for evidence of egg content removal by sucking arthropods. If an individual predator died during the assay, that replicate was discarded.

### Neonate trials

Following the same basic procedure, deli-cup predation arenas were used to assess potential predators of freshly hatched monarch neonates. Field collected *A. syriaca* or *A. incarnata* leaves were placed diagonally in the arena and a single neonate was placed on each leaf. Neonates were transferred to leaves with a fine-tipped paintbrush and observed under a microscope to ensure they were not damaged. Following placement, the caterpillars were left to acclimate for 10–20 minutes before a potential predator was added. Arenas were placed in the growth chamber, and neonate presence/absence and condition (alive or dead) was recorded at 48 h.

### Egg and neonate trials with ants

Because individual ants do not forage normally when displaced from the colony, ant predation was assessed by linking a predation arena (described above) to an ant colony held in the lab. Colonies of six different ant species and associated soil/litter were collected from locations in and around East Lansing, Michigan and placed in 20 × 21.5 × 11 cm (4 L) containers with Fluon (#2871C Insect-a-slip) applied to the inner top 2.5 cm to keep ants from escaping. Colonies were provided food and sugar water 2x per week and starved for 24 h prior to use in feeding trials to encourage foraging. As described above, monarch eggs or neonates were placed individually on field-collected *A. syriaca* or *A. incarnata* leaves in 473 mL deli-cups. We then connected ant colonies to the deli-cups using clear, flexible PVC tubing (0.64 cm ID, Model 702165 Home Depot; Fig. 1). We applied Fluon to the inner top 2.5 cm of each deli-cup to keep ants from escaping through the perforated lid. To initiate a trial, a wood coffee stirrer (3 mm width) was placed to connect the soil surface in the ant colony to the tube, allowing ants access to the test arena. The colony and arena assembly were then placed in the growth chamber and the egg/neonate presence was recorded at 48 h.

### Field observations of predation

Field studies were conducted in 2017–18 to identify factors influencing monarch oviposition and survival in different crop and non-crop habitats^21^. During these trials, 1581 sentinel monarch eggs were placed on milkweed stems and observed every 2 h for 24 h and again at 48 and 72 h. During these observations any incidences of predation on monarch eggs and larvae were recorded and the predator was identified to the lowest possible taxonomic level without disturbing its ongoing behaviour^21^. In addition, video surveillance cameras were used to determine the fate of 152 sentinel monarch eggs on milkweed in grassland habitats^50^. Here we use these observations to determine if taxa tested in our no-choice trials also prey on monarchs under field conditions.

## Data Availability

All data generated or analysed during this study are included in this published article.
